# Socio-ecological features other than sex affect habitat selection in the socially obligate monogamous Eurasian beaver

**DOI:** 10.1007/s00442-015-3388-1

**Published:** 2015-08-11

**Authors:** Sam M. J. G. Steyaert, Andreas Zedrosser, Frank Rosell

**Affiliations:** Department of Ecology and Natural Resource Management, Norwegian University of Life Sciences, 1430 Ås, Norway; Faculty of Arts and Sciences, Department of Environmental and Health Studies, Telemark University College, 3800 Bø, Norway; Institute of Wildlife Biology and Game Management, University of Natural Resources and Life Sciences, 1180 Vienna, Austria

**Keywords:** Habitat selection, Behavioral contexts, Monogamy, Eurasian beaver, *Castor fiber*

## Abstract

Habitat selection is a context-dependent mechanism, in which both the internal state as well as external factors affect the behavior and decisions of an individual. This is well known for polygamous mammals, which are typically sexually dimorphic, and often express great variability in behavior and habitat selection between individuals as well between the sexes. Among monogamous mammals, however, variability in habitat selection should be explained by group characteristics and the presence of offspring rather than by sex. We evaluated this hypothesis in a socially monogamous rodent, the Eurasian beaver (*Castor fiber*), in a saturated Norwegian population. For the first time in this species we applied GPS tracking devices (*N* = 22 adult beavers, in 15 territories, 2009–2013), and used resource selection functions (i) to document population-wide habitat selection and the importance of ‘territory’ therein, and (ii) to evaluate which socio-ecological factors explained potential individual differences in habitat selection. We found that variation in habitat selection was stronger between territories than between years or individuals nested by territory. We identified that family size and the presence of kits, but not sex, explained individual variation in habitat selection. Adults with kits and/or larger families tended to exhibit low risk-taking behavior (avoiding human-related variables such as roads, buildings, and agricultural land), and stayed close to their main lodge (parental care). Our results show that habitat selection is a context-dependent mechanism even in a species which expresses very little behavioral and morphological dimorphism.

**Electronic supplementary material:**

The online version of this article (doi:10.1007/s00442-015-3388-1) contains supplementary material, which is available to authorized users.

## Introduction

Optimal habitat selection in animals should provide for their immediate and long-term needs, without jeopardizing their survival (Manly et al. 2002). This implies that habitat selection should be context-dependent (Boyce et al. 2002; Luttbeg and Sih 2010), based on an individual’s internal state (e.g., reproductive status, sex, age, personality, etc.) and its external environment (e.g., climate, season, year, habitat quality, etc.) (Nathan et al. 2008). For example, vulnerability to predation can differ among individuals and can constrain habitat selection of more vulnerable individuals (Lima and Bednekoff 1998), reproductive strategies may diversify habitat selection and other behavior between the sexes or reproductive classes (Shuster and Wade 2003; Steyaert et al. 2013a, b, 2014), and group size and temporal variation in food availability may affect patterns in habitat selection (Johnson 1980; Fortin et al. 2009). Among mammals, such context-dependent habitat selection is more pronounced in polygamous than in monogamous species because of intersexual differences in behavior, morphology, and physiology. In polygamous species, males are typically larger than females, and have larger territories or home ranges or live spatiotemporally segregated from females, and paternal care is rare (Clutton-Brock 1989; Andersson 1994; Shuster and Wade 2003). In monogamous species, males and females are typically more similar in terms of body size and behavior, and paternal care is often essential to successfully raise offspring, especially in obligate monogamous species (Kleiman 1977; Gubernick and Teferi 2000; Reichard 2003). Consequently, one could expect that habitat selection is similar between the sexes in monogamous mammals.

Social monogamy implies a socio-spatial relationship in which a male and female stay together during at least one breeding season (Reichard 2003). Social monogamy is rare among mammals (~3–9 % of the species), and occurs mostly among canids, rodents, and primates (Kleiman 1977; Lukas and Clutton-Brock 2013). Socially monogamous species typically live in families and are territorial, implying that a dominant pair and their offspring share, maintain, and defend a single territory (Mathews 2002). This implies that habitat availability for family members is largely similar, if not identical. Therefore, patterns in habitat selection likely differ more strongly between individuals of different families than between family members. In addition to different habitat availability among territories, group size, habitat quality and resource availability, and the presence of offspring can also vary among territories, and thus should also influence patterns in habitat selection.

The socially obligate monogamous Eurasian beaver (*Castor fiber*) forms long-lasting breeding pairs, is territorial (Campbell et al. 2005), and both sexes contribute to territory maintenance (Rosell and Thomsen 2006) and parental care (Wilsson 1971). Beavers display little external sexual dimorphism, and adults of both sexes exhibit similar behaviour, although some division of labour has been observed (Wilsson 1971; Sharpe and Rosell 2003; Herr and Rosell 2004a; Rosell and Schulte 2004; Rosell and Thomsen 2006). Beavers are seasonal breeders, with a mating season from late January to early March, peaking around mid-February (Wilsson 1971). Kits are born around mid-May (Parker and Rosell 2001), and spend their first 6 to 8 weeks in the lodge (Wilsson 1971). Lactation lasts approximately 3 months (Zurowski et al. 1974). Male parental care includes socializing, allogrooming, transporting kits, providing food, huddling, and active territory defense (Sharpe and Rosell 2003). Family size typically ranges from two to six individuals (Rosell et al. 2006). Both sexes usually disperse at the age of 2 years (Hartman 1997), when beavers reach sexual maturity (Wilsson 1971). Beavers are herbivorous generalists, and feed on bark, leaves, and shoots of woody plants (preferably deciduous species), as well as on herbs, ferns, and aquatic vegetation (Wilsson 1971; Haarberg and Rosell 2006). Beavers exhibit a nocturnal lifestyle, typically remaining in their lodges during the day, although they may also frequently return to their lodges during nighttime (Rosell and Hovde 2001; Sharpe and Rosell 2003).

Here, we investigated habitat selection in the Eurasian beaver in relation to socio-ecological contexts. We hypothesized that (1) habitat selection at the population level (i.e., the statistical population of adult beavers in our study area) would be more pronounced between territories than between individuals, and that (2) on the individual level, family size and the presence of kits would have a greater influence on habitat selection than sex or season. We tested our hypotheses in a Norwegian beaver population based on global positioning system (GPS) tracking and resource selection functions.

## Materials and methods

### Study area

The study was carried out on the Straumen, Gvarv, and Sauar river systems in Telemark County, southeast Norway. The rivers are between 30 and 150 m wide, and represent comparable habitat systems. The rivers have a regulated slow water flow, and meander through a rolling landscape of pastures, arable land, and boreal forest. All three rivers empty into Lake Norsjø, and Lake Nome and Lake Bråfjorden are contained within the Straumen and Sauar rivers, respectively. Beavers do not build dams in the study area, as the rivers are large enough to make dam-building unnecessary (Herr and Rosell 2004b). The riverbanks consist mostly of bare soil and herbaceous vegetation. The forest is dominated by Norway spruce (*Picea abies*), Scots pine (*Pinus sylvestris*), and birch species (*Betula* spp.); however, deciduous species such as grey alder (*Alnus incana*), rowan (*Sorbus aucuparia*), and willow (*Salix* spp.) dominate forested riverbanks (Haarberg and Rosell 2006). The climate is cold and wet, with a mean annual temperature of 4.6 °C and annual precipitation of 790 mm. Elevations range from 15 to 70 m above sea level. Beavers resettled the study area in the 1920s, and the population is currently at carrying capacity (Rosell and Hovde 2001; Pinto et al. 2009). Today, hunting and trapping pressure is very low or nonexistent (Olstad 1937; Campbell et al. 2005). The Eurasian lynx (*Lynx lynx*) is the only natural predator, although it occurs at low densities (1996–2002: 0.2–0.4 lynx/100 km^2^) (Herfindal et al. 2005) and preys predominantly on species other than beavers (Odden et al. 2006).

### Trapping and sampling

We captured and marked adult beavers between 2009 and 2013, following the capture and handling protocol by Rosell and Hovde (2001). Captures were concentrated in spring (March–May) and autumn (August–October) of each study year. The study population is subject to a long-term monitoring program in which territory-specific social data (family size, annual reproductive success) is routinely collected (Campbell et al. 2012, 2013). We assumed that an animal was resident in a territory if we observed it several times within the same territory during its monitoring period. We are confident that we assigned the correct territory to each study individual, as we included only dominant individuals in this study, and virtually all GPS relocations were within their previously assigned territory. We classified animals into age classes (0 years = kit, 1 year = yearling, 2 years = sub-adults and ≥3 years = adult) based on their body weight (Rosell et al. 2010; Campbell et al. 2013). We assigned the adult residents of each sex as the dominant pair. If additional adult candidates were present within a territory, we verified dominance based on eventual dispersal of alternative candidates, body weight and size, and lactation (i.e., female nipple length >0.5 cm). We assumed that dominant individuals maintained their status until they disappeared or died, absent evidence to the contrary (Campbell et al. 2012).

We aimed to capture the dominant pair of a territory at least once during the study, and deployed a rechargeable archival µGPS receiver (24 g, ~20-day battery life) (model G1G 134A; Sirtrack, Havelock North, New Zealand, http://www.sirtrack.com). We also deployed a VHF transmitter (Reptile glue-on, series R1910; Advanced Telemetry Systems, Isanti, MN, USA), with a weight of 10 g, to track the animals for re-capture or to find lost devices. We glued the GPS and VHF units on the fur of the lower back with a two-component epoxy resin. We chose this part of the body for deployment, because it lies higher in the water surface when beavers swim and allows for better aerial sighting. We used a coarsely meshed polyester net to cover the units in order to prevent removal by the animal. We retrieved the GPS devices by recapturing the animals and cutting the device from the fur using a scalpel. We scheduled the GPS devices to fix one GPS location every 15 min between 1900 and 700 hours, given the nocturnal activity period of beavers (Sharpe and Rosell 2003). We discarded GPS relocations that were obtained on the days of capture and re-capture to avoid observer and capture-related bias in the data. Our capture and handling procedures, however, appear to have only minor behavioral effects, since beavers typically resume their normal activities within 15 min after their release (Sharpe and Rosell 2003). Long-term monitoring of our study animals does not affect reproductive success, lactation, or mothering ability (Ranheim 2004), and capturing and handling beavers has no significant effect on their body condition (252 beavers, 729 recaptures) (Farrell 2014). We screened the GPS data to improve spatial accuracy, according to Lewis et al. (2007). Successful GPS fixes were obtained only when a beaver was outside the lodge. All animal handling procedures were approved by the appropriate Norwegian authorities, the Norwegian Directorate for Nature Management and the Norwegian Animal Research Authority (Forsøksdyrutvalget, NARA/FDU).

### Data analyses

We used resource selection functions (RSFs) to (i) identify habitat selection on the population level and determine the importance of ‘territory’, ‘individual’, and ‘year’ therein, and to (ii) evaluate how sex, the presence of kits, and family size influence habitat selection on the individual level. RSFs contrast habitat ‘use’ with habitat ‘availability’, which is typically represented by a set of animal relocations (GPS data) and a set of random points, respectively. Subsequently, the use and availability data can be linked to spatial data layers (land cover type, slope steepness, etc.) in a geographical information system, and statistical methods (e.g., logistic regression) can be used to determine which and how landscape characteristics influence resource selection (Aarts et al. 2012). For details on the RSF methodology, see Lele et al. (2013). We sampled habitat use and habitat availability in a 1:1 ratio, within the 100 % minimum convex polygon (MCP) estimated territory of each individual beaver during the period of monitoring.

For each location, we derived the following landscape variables from a continuously updated digital topographic map (Felles KartDatabase, FKB data Geovekst, http://www.kartverket.no/) of the study area: land cover [‘forest’, ‘build-up’ (land covered with buildings), ‘water’, ‘agriculture’, ‘mire’, ‘other’], slope steepness, river or lake associated (if the nearest water body to a certain location was a lake or a river, we defined lakes and river based on their administrative classification), distance to the nearest water body (for locations on land), distance to the nearest land (for locations in the water), and distance to roads, buildings, and to the main lodge. We considered these landscape variables as fixed effects in mixed effect regression models.

For habitat selection on the population level, we used ‘use’ vs. ‘availability’ as the binomial response variable, and included ‘beaver ID’ nested in ‘territory ID’ as random factors on the intercept. Because climate and climatic variation can influence recruitment in beavers (Campbell et al. 2012), we included ‘year’ as a random factor. We formulated six candidate models a priori (Electronic Supplementary Material Table A1), and used second-order bias-corrected Akaike information criteria (AIC_c_) differences (ΔAIC_c_) and weights (AIC_wt_) to select the most parsimonious model (Anderson 2008). The variables ‘land cover’ and ‘river/lake associated’ were considered as categorical variables, whereas the ‘distance to’ variables were considered as continuous variables. We considered models with ΔAIC_c_ values >2 as inconclusive. We evaluated the relative importance of each model term of the most parsimonious model based on ΔAIC_c_ scores after systematically including/excluding model components. We validated the most parsimonious model using a tenfold cross-validation procedure following Boyce et al. (2002). We chose 0.6 as the threshold level for evaluating collinearity among explanatory variables. We multiplied the parameter estimates of the ‘distance to the nearest…’ variables by −1 to facilitate interpretation, with positive values indicating selection and negative values indicating avoidance.

For habitat selection on the individual level, we aimed to explain variance in habitat selection among individuals in relation to socio-ecological factors. Therefore, we created individual-based RSFs with respect to a fixed set of landscape variables; all ‘distance to the nearest…’ variables and the land cover classes that occurred in all 100 % MCP territory. For each beaver, we sampled use/availability in a 1:1 ratio, and within its 100 % MCP territory. The parameter estimates (*β*) and standard errors (*σ*) for each model variable indicate whether a certain variable is selected for, selected against, or is of relative unimportance in an individual’s habitat selection, and can be considered as selection coefficients for individual behavioral responses (Compton et al. 2002). We summarized these behavioral responses of the individual RSFs, and linked them with data of each individual beaver, year of monitoring, season of monitoring (spring/autumn), sex, the presence of kits (yes/no), territory size (100 % MCP estimated territory size based on valid GPS relocations), and family size (total number of beavers in the colony). We evaluated the model fit of the individual RSFs using the dispersion parameter (residual deviance/degrees of freedom, should be close to 1). For each individual, we obtained behavioral responses for ‘slope’, distance to ‘roads’, ‘buildings’, ‘water’, ‘land’, and ‘lodges’, and land cover types ‘agriculture’, ‘forest’, ‘mire’, and ‘water’. We did not obtain behavioral responses for ‘river or lake associated’ because of singularities, or for the land cover types ‘other’ and ‘build-up’ because of their absence in some of the territories. We used these behavioral responses, as well as territory size, as response variables in linear regression models, and considered ‘season of monitoring’ and the social factors ‘sex’, ‘family size’, ‘presence of kits’, and the interaction ‘presence of kits × sex’ as explanatory variables. We have not included the variable ‘year’ at this stage of the analysis, because of singularities and a potentially confounding relationship with ‘beaver ID’. For the behavioral responses to the land cover classes, we also included the availability of a given land cover type in each beaver's territory to account for a potential functional response (Mysterud and Ims 1998). For these post hoc analyses, we ran all possible model combinations, and selected the most parsimonious model based on the ΔAIC_c_ and AIC_cw_ scores (Burnham and Anderson 2002). Based on the principle of parsimony, and to avoid including ‘pretending variables’ in the model results, we selected the simplest model within the ΔAIC_c_ range of 0–2 (Anderson 2008). We used a linear mixed-effect regression model with the number of valid daily GPS relocations per individual as the response variable, ‘sex’ as a fixed effect, and ‘beaver ID’ as a random effect on the intercept to test whether the number of valid GPS relocations differed between the sexes, and we used a linear model to evaluate the relationship between the total number of days that an individual was monitored and ‘sex’. We validated the most parsimonious models by plotting the model residuals versus the fitted values (Zuur et al. 2009). We used R 2.15.0 software for all statistical analyses (R Development Core Team 2013).

## Results

We obtained GPS relocation data for 22 dominant beavers (12 males, 10 females) among 15 territories (Table 1). With the exception of one male (Erlend), all beavers were monitored during a single year and a single season. We managed to simultaneously monitor the dominant pair of a territory on five occasions (Territory B1, spring 2010; LP, autumn 2010; P0, autumn 2010; P1, autumn 2013; P3b, autumn 2010) (Table 1). Beavers were monitored on average for 13.5 ± 4.5 (8–25) days before the GPS units were retrieved. The average number of successful GPS fixes per individual was 361 ± 158 (range 158–689) (Table 1). There was no difference between the sexes in either the number of daily GPS relocations per individual or the number of monitoring days per individual (daily relocations: *β* = −1.3, *σ* = 2.69, LL = −6.59, UL = 3.99, ΔAIC_c_ = −1.93; monitoring days: *β* = −2.2, *σ* = 2.96, LL = −8.00, UL = 3.61, ΔAIC_c_ = 2.06). No collinearity was apparent in the data.

**Table 1:** Meta-data of the dominant GPS-marked beavers used in this study in Telemark County, Norway, from 2009 to 2013

Beaver	Territory	Sex	*N*_pos_	*N*_day_	Year	Season	# kits	Family size
Leslie	B1	F	679	19	2010	s	0	2
Andreas	B1	M	275	10	2010	s	0	2
Moritz	B2	M	168	8	2010	a	0	3
Hazel	G	F	682	25	2010	s	0	8
Paddy	G	M	375	12	2012	s	3	6
Lasse	L2	M	325	11	2011	s	1	5
Loran	L4a	M	412	13	2009	a	0	3
Maud	L6a	F	392	12	2009	a	0	2
Bram	L6a	M	158	8	2011	s	0	3
Ida	LP	F	383	14	2010	a	2	5
Kjartan	LP	M	216	9	2010	a	2	5
Jodie	N1	F	388	13	2012	s	1	5
Hanna Synnøve	P0	F	257	12	2010	a	1	4
Jan Marc	P0	M	384	14	2010	a	1	4
Live	P1	F	249	21	2013	a	1	5
Manuel	P1	M	297	11	2013	a	1	5
Apple	P2a	F	689	18	2013	a	0	5
Leigh	P2a	F	180	9	2010	a	1	4
Moses	P2b	M	279	14	2010	a	2	5
Christina	P3a	F	459	19	2010	a	0	3
Erlend	P3b	M	446	15	2010	a	0	2
Erlend	P3b	M	437	17	2013	s	0	2
Horst	P4	M	171	8	2010	a	1	3

*Territory* territory identifier, sex (*m* male, *f* female), *N*_*pos*_ number of valid GPS relocations per individual, *N*_*day*_ number of monitoring days per individual. *Year* year of monitoring, Season (*s* spring, *a* autumn), *No. kits* the number of kits in the family, *Family size* the total number of family members in a given territory during the period of monitoring

### Habitat selection on the population level

The full model was the most parsimonious (AIC_cw_ = 1) of the six a priori defined candidate models. All other candidates were inconclusive (all ΔAIC_c_ scores >85). The strongest predictors of beaver habitat selection were the distance to water while a beaver was on land and the distance to land while a beaver was in the water (Table 2). Beavers generally selected their habitat relatively close to the river bank (median, mean, and 95th percentile for GPS locations on land were 15, 24.8, and 77 m, respectively, and 13, 17, and 49 m for GPS locations in the water, respectively) (Electronic Supplementary Material Fig. A1). Land cover type was an important determinant of beaver habitat selection. With build-up areas as a reference, water bodies, mires, and forests were selected for, whereas agricultural areas and other land cover types were neither selected for nor against (Table 2). Relative to random locations, beavers selected areas close to roads and far from their lodges (Table 2). They generally preferred areas in or close to rivers over areas in or close to lakes, and selected for gentle slopes (Table 2). Buildings had no apparent effect on habitat selection, and excluding ‘distance to the nearest building’ from the full model would have resulted in a slightly better model fit (Table 2). Habitat selection varied considerably more (~6 times) between territories (random effect variance = 0.118) than between individual beavers (random effect variance = 0.0196) or years (random effect variance = 0.0198). The full model had good predictive accuracy (tenfold cross-validation coefficient = 0.89).

**Table 2:** Fixed effects of the most parsimonious model for estimating population-wide habitat selection of Eurasian beavers in Telemark, southern Norway (2009–2013)

Model term	*β*	*σ*	LL	UL	ΔAIC_c_
Distance to					
Intercept	0.6574	0.2269	0.213	1.102	
Road	0.0011	0.0003	0.001	0.002	72.1
Lodge	−0.0005	0.0001	−0.0007	−0.0003	17
Land	0.0441	0.0014	0.041	0.047	1723.5
Building	0.0001	0.0003	−0.0005	0.0007	−1.68
Water	0.0283	0.0008	0.027	0.03	2056.4
Slope steepness	−0.0137	0.003	−0.02	−0.008	66.55
Habitat type					
River vs. lake	0.3525	0.0607	0.234	0.471	59.27
Water vs. build-up	0.8927	0.1861	0.528	1.257	315.5
Mire vs. build-up	0.6002	0.218	0.173	1.027	
Forest vs. build-up	0.6044	0.1815	0.249	0.96	
Agriculture vs. build-up	−0.3545	0.1882	−0.723	0.014	
Other vs. build-up	−0.0188	0.199	−0.409	0.371	

Large values indicate high relative importance of a certain model term. LL and UL represent the lower and upper 95 % confidence levels around the parameter estimate (*β* ± 1.96 × *σ*). Positive *β* values indicate selection for, whereas negative values indicate selection against

*β* parameter estimate, *σ* standard error, *ΔAIC*_*c*_ Akaike information criterion difference calculated separately for each model term with respect to the full model

### Habitat selection on the individual level

The individual-based RSFs had dispersion parameters between 0.72 and 1.19, indicating good model fit. Territory size and selection for ‘slope steepness’, the land cover type ‘mire’, and selection in relation to ‘distance to water’ was not related to social characteristics in beavers (Table 3). ‘Family size’ was included in the top-ranked models for 3 of 11 behavioral types. Adults of larger families (~8 individuals) selected their habitat closer to the lodge than adults of smaller families (~2 individuals, Table 1) (Table 3, Electronic Supplementary Material Fig. A2–4). Adults of larger families selected their habitat further from the bank when in the water, and avoided areas close to buildings more strongly than adults from smaller families (Table 3, Electronic Supplementary Material Fig. A2–4). Families with kits avoided agricultural areas and roads to a greater degree than families without kits (Table 3). In general, beavers showed a negative functional response for the land cover types ‘water’ and ‘forest’: the scarcer these land cover types, the more strongly individuals selected for them (Table 3). Sex and season were never included in the most parsimonious models (Table 3). No heteroskedasticity was present in the models.

**Table 3:** Most parsimonious model outcomes that relate selection behaviors and territory size (response variable) to socio-ecological factors (‘season of monitoring’, ‘sex’, ‘family size’, ‘presence of kits’, and the interaction ‘sex × presence of kits’)

Response variable	Model term (s)	*β*	*σ*	LL	UL	ΔAIC_c_	*R* ^2^
Slope steepness	–	–	–	–	–	–	–
Territory size	–	–	–	–	–	–	–
Land cover type: ‘mire’	–	–	–	–	–	–	–
Land cover type: ‘agriculture’	Presence of kits	−0.44	0.09	−0.616	−0.264	12.1	0.17
Land cover type: ‘water’	Availability of water	−0.595	0.201	−0.989	−0.201	5.4	0.30
Land cover type: ‘forest’	Availability of forest	−1.051	0.247	−1.535	−0.567	11.7	0.10
Distance to the nearest road	Presence of kits	−0.004	0.002	−0.008	0.000	4.0	0.25
Distance to the nearest lodge	Family size	0.001	0.0004	0.000	0.002	5.2	0.29
Distance to the nearest land	Family size	−0.007	0.002	−0.011	−0.003	10.7	0.44
Distance to the nearest building	Family size	−0.002	0.001	−0.004	0.000	3.5	0.23
Distance to the nearest water	Family size	0.005	0.002	0.001	0.009	0.8	0.13

LL and UL represent the 95 % confidence levels around the parameter estimate (*β* ± 1.96 × *σ*). Note that ΔAIC_c_ values <2 indicate uninformative model terms*. Β* parameter estimate, *σ* standard error, *ΔAIC*_*c*_ Akaike information criterion difference values relative to the second most parsimonious model

## Discussion

Our results support hypothesis (1), because habitat selection varied considerably more (~6 times) among territories than among individual beavers or years. We also found support for hypothesis (2, as family size and the presence of kits best explained variation in habitat selection and behavior, and that habitat selection did not differ between the sexes. Concurrent with the theory (Mysterud and Ims 1998), beavers also exhibited a functional response for forest and water: the scarcer these resources, the more strongly they were selected for.

### Habitat selection on the population level

The general patterns of habitat selection was consistent with expectations for an aquatic rodent: a very strong link to the river bank or lakeshore, forest and mires or swamps, and a tendency to avoid steep surfaces (Collen and Gibson 2000). We suggest two possible explanations why beavers selected for areas far from their lodges. Central-place foraging implies that individuals must bring their food items to a central place for processing, storage, or consumption (Orians and Pearson 1979). For beavers, such a central place implies the river bank or lakeshore, as well as their lodge. Beavers generally move out of the water to select their food items on land, and transport them back to the water to consume directly or to the lodge to feed their offspring or to store for later use (Schoener 1979; Jenkins 1980; McGinley and Whitham 1985; Basey et al. 1988; Fryxell and Doucet 1991; Haarberg and Rosell 2006). However, in territories that have been stable and maintained over several generations, food depletion near the main lodge may force beavers to select their resources far from the lodge (Fryxell and Doucet 1991; Collen and Gibson 2000). Secondly, territory maintenance (scent marking), patrol, and defense should be most concentrated in areas where the probability of intrusion is highest, at the up- and downstream border zones (Rosell et al. 1998; Herr and Rosell 2004a). These border zones are typically far from the main lodge.

Surprisingly, we found that beavers generally tended to select areas close to roads. We suggest that beavers, like many omnivores and other herbivores, are attracted to nutritious herbaceous roadside vegetation (Forman and Alexander 1998). Distance to roads was not spatially correlated with distance to water or other landscape variables, suggesting that preference for roads was an independent factor. Investigation of the random model terms and individual-based RSFs, however, suggested that habitat selection was more complex, and varied among individuals according to their socio-ecological situation.

### Habitat selection on the individual level

Family size and the presence of kits were important socio-ecological factors that affected habitat selection on the individual level. We suggest that adult beavers with large families and/or kits alter their habitat selection according to the parental care required to successfully raise their offspring. For example, adults with kits should return to the lodge more frequently to feed, or to huddle and provide body warmth to their kits, than beavers without kits (Sharpe and Rosell 2003; Sun 2003). Seasonal variation appeared to be relatively unimportant in habitat selection behavior among beavers.

Life history theory predicts that, in order to increase offspring survival, individuals with dependent offspring should be less willing to expose themselves to risk than individuals without dependent young (Stearns 1989; Lima and Dill 1990). Our results concur with this prediction, because we observed that adult beavers of larger families and/or with kits were more likely to avoid areas with a high risk of meeting humans (agricultural areas, and roads) than adults of smaller families or without kits. Predation, hunting, and exploitation can have long-lasting behavioral and evolutionary effects on wildlife populations (Creel et al. 2007; Proaktor et al. 2007; Zedrosser et al. 2011). Even if human hunting and natural predation are currently low in our study population, such lasting risk avoidance effects are not surprising. Past research has documented that beavers decreased their foraging activity and scent-marking behavior in the presence of experimentally applied predator [wolf (*Canis lupus*), brown bear (*Ursus arctos*), river otter (*Lutra lutra*), red fox (*Vulpes vulpes*), and lynx] odors (Rosell and Czech 2000; Rosell and Sanda 2006). Behavioral differences between individuals of different reproductive status or presence of offspring should be expected, considering that paternal care is an extremely important factor in shaping the general ecology of a species (Andersson 1994). Such behavioral differences have been documented in a range of species, including those with both monogamous and polygamous mating systems. For example, spatiotemporal behavior of wolves is concentrated around a central place (den or rendezvous sites) during the summer, when the pups are fully dependent on their pack mates, whereas they have a more nomadic lifestyle when no dependent young are present (Boitani 2003). Similarly, female brown bears with dependent offspring alter their habitat selection compared to lone females in order to reduce infanticide risk (Steyaert et al. 2013a, b).

### The absence of ‘sex’ in the models

The factor ‘sex’ was never included in the most parsimonious models. Our results concur with previous research on both Eurasian and North American beavers (*C. canadensis*), which suggests that the two sexes of adult dominant beavers exhibit very similar behavior in terms of use of space within a given territory (Hodgdon and Lancia 1983; Busher and Jenkins 1985; Sharpe and Rosell 2003; Herr and Rosell 2004b; Busher 2007).

Behavioral variation between the sexes is common in polygamous, sexually dimorphic species (Ruckstuhl and Neuhaus 2005), and is generally explained by different parental investments by males and females. Females can optimize their reproductive success by safeguarding the survival and primary needs of their dependent offspring, whereas the behavior of the males is typically not constrained by the presence of young. This is referred to as the ‘reproductive strategy hypothesis’ in the sexual segregation literature (Bowyer 2004; Ruckstuhl and Neuhaus 2005; Ruckstuhl 2007; Main 2008), and evidence for this hypothesis has been documented in a range of ungulates and carnivores (Ruckstuhl and Neuhaus 2005; Main 2008; Steyaert et al. 2013a, b).

Theory predicts that the two sexes in monogamous species are less dimorphic in size and behavior than those in polygamous species (Kleiman 1977). However, sex differences can never completely disappear (Magurran and Garcia 2000). For example, lactation is an extremely costly process for females (Millar 1977), and influences use of space and behavior in many—if not all—mammals (Cost et al. 2014). In beavers, male investment in the family is mostly allocated to construction behavior, territory maintenance (scent marking and defense) (Rosell and Thomsen 2006), and alarm behaviors (Busher 2007). We could not detect such a difference between the sexes. Possible explanations for this might be that males and females are, indeed, very similar in terms of behavior and use of space, implying that males play an important role in parental care (Sun 2003), that behavioral differences such as nursing are predominantly expressed in the lodge (outside direct view/lost GPS connection), or that such differences are simply not detected with the use of GPS data.

## Conclusions

We found that differences in habitat selection behavior in beavers were more pronounced between than within territories. These differences were related to family size and the presence of kits, but not to sex. In addition, beavers showed a functional response to certain habitat types, which indicates that part of the behavioral variation among the territories can be explained by variation in habitat availability. The behavior of adult beavers reflected strategies to optimize reproductive success; in the presence of kits and/or larger families, adults tended to exhibit low risk-taking behavior (avoiding human-related variables such as roads, buildings, and agricultural land), and stayed relatively close to the main lodge (suggesting parental care by both sexes). Our results add to the growing body of evidence that habitat selection behavior is dependent on an organism’s internal and external contexts, even in a species which expresses very little behavioral and morphological dimorphism.

## Electronic supplementary material

Supplementary material 1 (DOCX 132 kb)
