# Supplementary material for: Socio-ecological features other than sex affect habitat selection in the socially obligate monogamous Eurasian beaver
Source: Oecologia. 2015 Aug 11;179(4):1023–32. doi: 10.1007/s00442-015-3388-1 (PMC4630256; doi:10.1007/s00442-015-3388-1)

**Electronic Supplementary Material**

Table A1. Model diagnostics of the six *a priori* defined candidate models to estimate population wide resource selection of Eurasian beavers in Telemark, southern Norway (2009-2013). ΔAIC_c_ = Akaikes Information Criterion difference, AIC_wt_ = Akaikes Information Criterion model weight. ✔ indicates whether or not a variable was included in a candidate model.

| **Model** | **Distance to the nearest:** | | | | | **Slope** | **River/Lake** | **Land cover** | **ΔAIC_c_** | **AIC_wt_** |
| --- | --- | --- | --- | --- | --- | --- | --- | --- | --- | --- |
|  | **Road** | **Lodge** | **Land** | **Water** | **Building** |  |  |  |  |  |
| Full | ✔ | ✔ | ✔ | ✔ | ✔ | ✔ | ✔ | ✔ | 0 | 1 |
| Expert |  | ✔ | ✔ | ✔ |  | ✔ | ✔ | ✔ | 85.25 | 0 |
| Bank |  |  | ✔ | ✔ |  | ✔ |  |  | 304.18 | 0 |
| Null |  |  |  |  |  |  |  |  | 5362.7 | 0 |
| Human | ✔ |  |  |  | ✔ |  |  |  | 5353.96 | 0 |
| Terrain |  |  |  |  |  | ✔ | ✔ | ✔ | 3875.61 | 0 |

Figure A1: Frequency distributions of GPS relocations of beavers in relation to distance to land (when being in the water), and distance to water (when being on land).


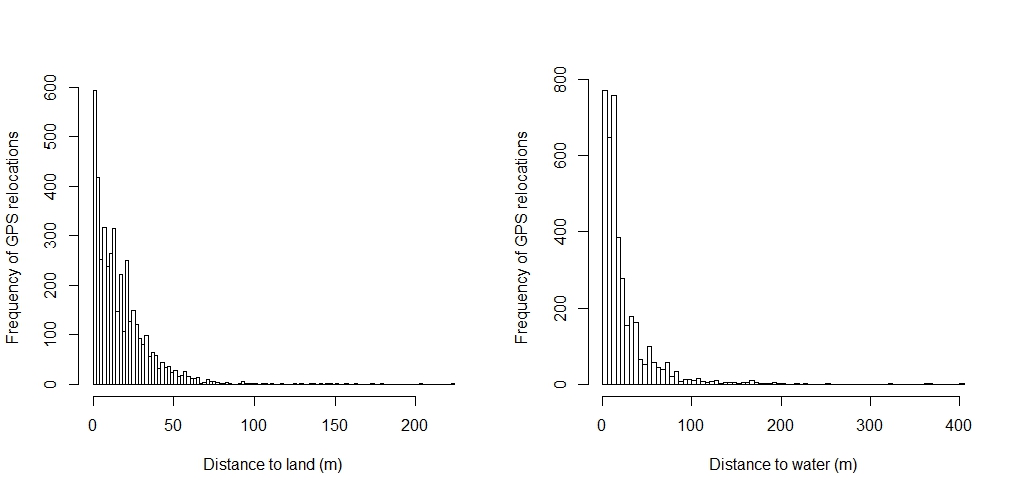


Figure A2: Relationship between family size and selection behavior for ‘Distance to the nearest lodge’.


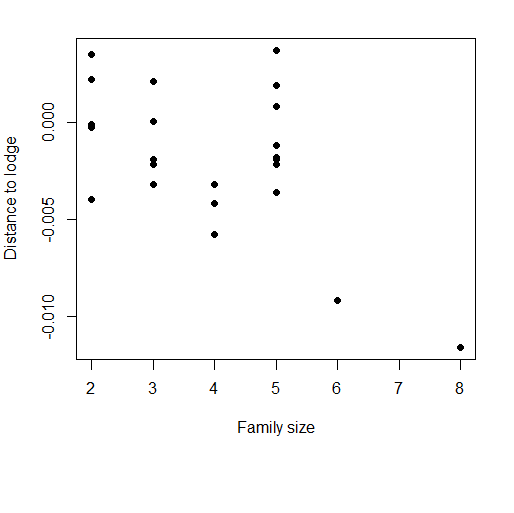


Figure A3: Relationship between family size and selection behavior for ‘Distance to the land’.


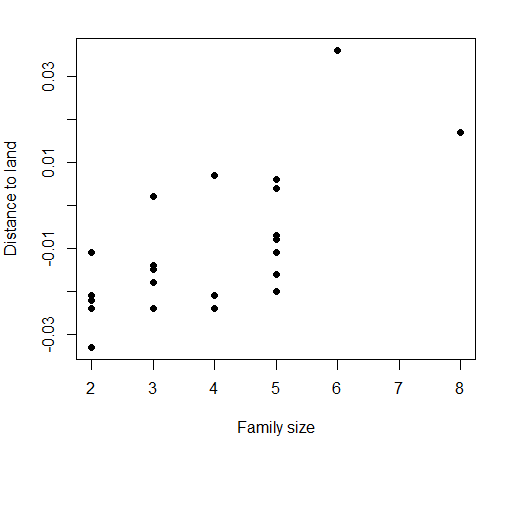


Figure A4: Relationship between family size and selection behavior for ‘Distance to the nearest building’.


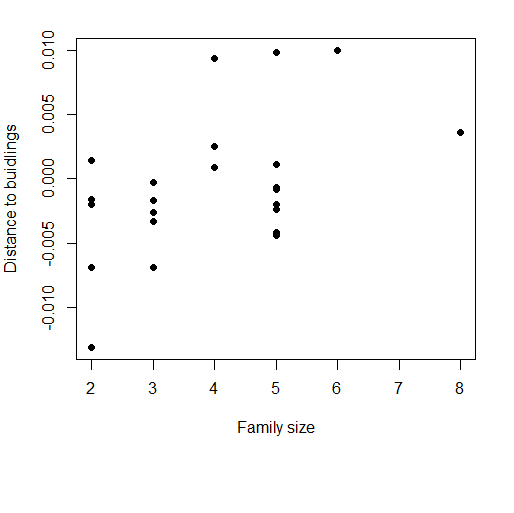

Supplement: Supplementary file 1 — Supplementary material 1 (DOCX 132 kb) [file 442_2015_3388_MOESM1_ESM.docx]
